# Direct regulation of *p53* by *miR-142a-3p* mediates the survival of hematopoietic stem and progenitor cells in zebrafish

**DOI:** 10.1038/celldisc.2015.27

**Published:** 2015-09-15

**Authors:** Xinyan Lu, Yonglong Wei, Feng Liu

**Affiliations:** 1 State Key Laboratory of Membrane Biology, Institute of Zoology, Chinese Academy of Sciences, Beijing, China

**Keywords:** *miR-142a-3p*, *p53*, HSCs, HSPCs, survival

## Abstract

Hematopoietic stem and progenitor cells have the capacity to self-renew and differentiate into all blood cell lineages, and thus sustain life-long homeostasis of the hematopoietic system. Although intensive studies have focused on the orchestrated genetic network of hematopoietic stem and progenitor cell specification and expansion, relatively little is known on the regulation of hematopoietic stem and progenitor cell survival during embryogenesis. Here, we generated two types of *miR-142a-3p* genetic mutants in zebrafish and showed that the loss-of-function mutants displayed severe reduction of hematopoietic stem and progenitor cells. Further analysis showed that the diminished proliferation and excessive apoptosis in *miR-142a-3p* mutants were attributed to the increased *p53* signaling. Mechanistically, we demonstrated that *miR-142a-3p* directly targets *p53* during hematopoietic stem and progenitor cell development, and the hematopoietic stem and progenitor cell survival defect in *miR-142a-3p* mutants could be rescued by loss of *p53*. Therefore, our work reveals the significance of the *miR-142a-3p*-*p53* pathway in controlling hematopoietic stem and progenitor cell survival, and thus advances our understanding of the role of *p53* in vertebrate hematopoiesis.

## Introduction

Hematopoiesis in vertebrate is initiated in two distinct and successive processes termed primitive and definitive waves [1]. Hematopoietic stem and progenitor cells (HSPCs), which are generated from definitive hematopoiesis via a specific endothelial-to-hematopoietic transition process [2–5], possess the capacities of self-renewal and differentiation into erythroid, myeloid and lymphoid lineages to sustain blood cells during embryogenesis and adulthood. The emergence of HSPCs is tightly regulated by signaling pathways (Notch, Hedgehog, BMP, Wnt and pro-inflammatory signals) [6–10], transcription factors (Runx1, Cbfb and Scl) [11–13], G-coupled protein receptor 56 [14] and epigenetic regulators [15, 16].

MicroRNAs are reported to be highly expressed in hematopoietic tissues including bone marrow [17] and emerging evidence has shown that they are implicated in hematopoiesis in different organisms [18–20]. In mouse, *miR-125* is highly expressed in hematopoietic stem cells (HSCs) to expand HSC numbers *in vivo* [21]. *In vitro*, mouse fibroblasts were reprogrammed into hemogenic endothelial cells that differentiated into CD45^+^ cKit^+^ cells (HSPCs) between day-20 and day-35 culture; in these HSPCs, the highest enrichment of microRNAs including *miR-142* was observed [22]. In Xenopus, *miR-142-3p* has an essential role in establishing the mesodermal lineage that contributes to both HSC emergence and vasculogenesis [23]. In zebrafish, miR-142-3p modulates neutrophil development by controlling its maturation [24]. MiR-142 is also an essential regulator of lymphocyte ontogenesis and megakaryopoiesis [20, 25]. In our previous study, suppression of *miR-142a-3p* by antisense morpholinos shows that HSPC development is impaired when *miR-142a-3p* is knocked down. Further microarray data and functional assays demonstrate that *irf7* acts as a direct target of *miR-142a-3p* and that the *miR-142a-3p-irf7* signal pathway regulates the formation and differentiation of definitive HSPCs possibly through the inflammatory signaling [26]. Another group has reported that ectopic expression of miR-142-3p leads to the reduction of primitive erythrocyte progenitor cells and HSCs in zebrafish [27]. Thus, the role of zebrafish *miR-142a-3p* in definitive hematopoiesis remains elusive, largely because of different methods used by different groups.

P53, a tumor suppressor involved in many disorders including hematological malignancy [28], can activate DNA repair proteins when DNA is under sustained damage, leading to apoptosis and G1/S cell cycle arrest [29–31]. *p53* is a critical transcription factor for cell cycle regulation in hematopoietic cells and is involved in the quiescence, self-renewal, senescence and apoptosis of HSCs [32, 33]. HSCs from hypermorphic *p53* mice, which constitutively activates P53, fail to expand [34] and HSC self-renewal in *p53*^−/−^ mice is enhanced [35]. In the zebrafish Diamond-Blackfan model, the downregulation of erythroid marker *gata1* is modulated by *p5*3 [36]. Intriguingly, loss of *p53* facilitates reprogramming murine fibroblasts to hematopoietic progenitor cells and *p53*^−/−^ reprogrammed cells efficiently generated erythroid, megakaryocytic, myeloid, and lymphoid lineages [37]. In addition, suppression of *p53* can exert hematopoietic rescue effect on genetic alterations including *mysm1* deficiency [38], and *p53*^−/−^ HSCs are more resistant to irradiation [32]. Taken together, *p53* may be closely linked to the cell cycle and apoptosis and thus critical for the survival and stemness maintenance of HSPCs. *Gfi-1*, *Necdin* and *P21* were the downstream targets of *P53* in mediating HSC quiescence and *Puma* was responsible for HSC apoptosis [28, 29]. However, the upstream regulator(s) of *p53* governing HSC development is largely unclear.

In this work, we demonstrate that *p53* is a direct target of *miR-142a-3p* during zebrafish definitive hematopoiesis, and the hematopoietic defects in *miR-142a-3p* mutants can be rescued by loss of *p53*, which deepens our understanding of the role of *p53* in mediating *miR-142a-3p* function in HSPC development in zebrafish.

## Results

### Generation of *miR-142a-3p* mutants by TALENs and CRISPR/Cas9

**Figure 1:**
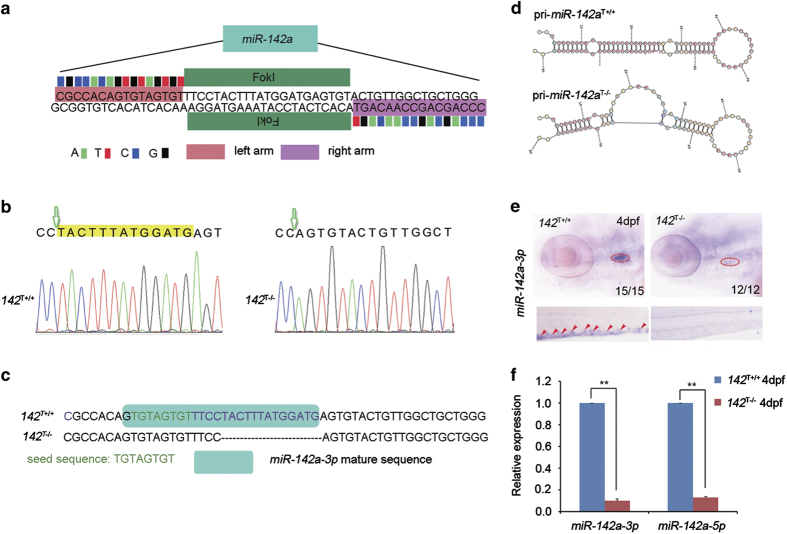
Generation of *miR-142a* mutants by TALENs. (**a**) Schematic diagram showed the TALEN target sites at the *miR-142a* locus. (**b** and **c**) Indel mutations induced by TALENs showed a 13-base-pair deletion in the *miR-142a* locus. Green arrow shows the initiation of the mutation. (**d**) Predicted RNA secondary structure of wild-type and mutated pri-*miR-142a* by TALENs using the RNA Structure Software (version 4.6, Rochester, NY, USA). (**e**) Expression of *miR*-*142a-3p* was absent in *142*^T−/−^ embryos at 4 days post-fertilization (dpf) by WISH. Red dashed circles mark the expression of *miR-142a-3p* in the thymus and red arrowheads mark the expression of *miR-142a-3p* in the CHT. (**f**) qPCR demonstrated that the expression of *miR-142a-3p* and *miR-142a-5p* was nearly undetectable in *142*^T−/−^ embryos at 4 dpf. The expression of *miR-142a-3p* and *miR-142a-5p* was normalized to *U6* (mean±s.d., *n*=3, **P<0.01).

In order to substantiate our previous findings that loss of *miR-142a-3p* leads to decreased HSPCs [26], both TALENs and CRISPR/Cas9 were used to generate genetic mutants of *miR-142a*. First, we designed a pair of TALEN arms that target two sites inside of the *miR-142a* gene (Figure 1a). The TALEN mRNAs were injected into WT embryos (F0) and after genotyping of F2 embryos, a mutant, named *142*^T−/−^, was identified. *142*^T−/−^ embryos were born at the expected Mendel ratio, and they could develop into fertile adults. According to the sequencing result, 13 base pairs were deleted in the locus of *the miR-142a* gene (Figure 1b and c) and this disrupted the secondary structure of *miR-142a* as well as the formation of mature *miR-142a-3p* (Figure 1d). Further analysis revealed that the expression of *miR-142a-3p* in both the thymus and caudal hematopoietic tissue region was undetectable (Figure 1e). Consistently, real-time quantitative PCR (qPCR) result demonstrated that *miR-142a-3p* and another member of *miR-142a*, *miR-142a-5p*, were severely decreased (Figure 1f), indicating the loss-of-function of *miR-142a-3p* and *miR-142a-5p*.

To generate a large deletion of the *miR-142a* gene by CRISPR/Cas9, a pair of gRNAs flanking the *miR-142a* locus was designed and co-injected into zebrafish WT embryos with *Cas9* mRNA (Supplementary Figure S1a). After F2 screening, a *miR-142a* mutant was identified, named *142*^C−/−^, and the sequencing result displayed a 986-base-pair deletion in the locus of *miR-142a*, which was able to be employed to distinguish the WT siblings and mutants by PCR (Supplementary Figure S1b). Impaired secondary RNA structure of *miR-142a* and failure to form the mature *miR-142a-3p* also occurred in *142*^C−/−^ embryos (Supplementary Figure S1c and d). Whole-mount *in situ* hybridization (WISH) result demonstrated that *miR-142-3p* was absent in both the thymus and caudal hematopoietic tissue region in *142*^C−/−^ embryos (Supplementary Figure S1e), suggesting a loss-of-function of *miR-142a-3p*.

Taken together, two zebrafish genetic mutants of *miR-142a* were generated in which *miR-142a-3p* was disrupted and thus facilitated functional investigation of *miR-142a* in zebrafish hematopoiesis.

### Loss of *miR-142a-3p* leads to decreased HSPCs in zebrafish

Previous studies implicate that loss of miR-142a-3p leads to decreased erythrocytes as well as vascular defects [39, 40]. Thus, we examined whether *miR-142a-3p* mutants display such developmental defects. With a somitic marker *myod* to define specific developmental stages, WISH results showed that primitive hematopoiesis including erythroid progenitors (labeled by *scl* and *gata1*) and myeloid cells (labeled by *lyz* and *pu.1*) was unaffected in *142*^T−/−^ embryos at 24 and 36 hours post-fertilization (hpf), respectively (Supplementary Figure S2a and b). Moreover, vascular development was examined and artery–vein specification was relatively normal as expression of arterial markers *dll4*, *dltC* and *efnb2a* and venous markers *flt4* and *msr* at 26 and 36 hpf was unaltered (Supplementary Figure S2c and d). Real-time PCR results also displayed normal expression of *dll4* and *flt4* in *142*^T−/−^ (Supplementary Figure S2e). Taken together, primitive hematopoiesis and vascular development were normal in *142*^T−/−^ embryos.

**Figure 2:**
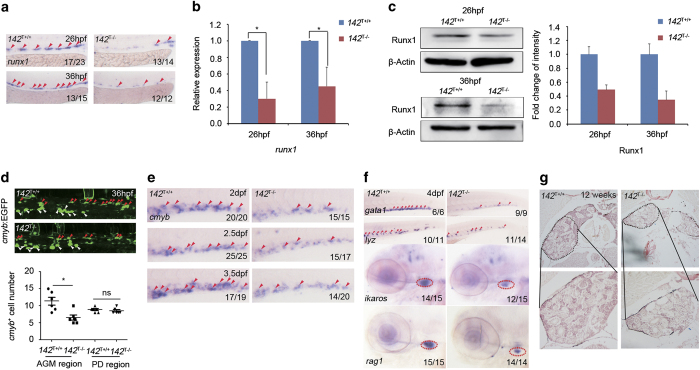
*MiR-142a-3p* is essential for HSPC emergence. (**a**) *142*^T−/−^ embryos showed decreased expression of HSPC marker *runx1* at 26 and 36 hpf by WISH. (**b**) *142*^T−/−^ embryos displayed decreased expression of HSC marker *runx1* at 26 and 36 hpf using qPCR. The expression of *runx1* was normalized to *β-actin* (mean±s.d., *n*=3, **P<0.05). (**c**) Runx1 was decreased in *142*^T−/−^ embryos at 26 and 36 hpf by western blot analysis (left panel) and the quantification (right panel). The protein level of Runx1 was normalized to β-Actin. (**d**) The number of EGFP-labeled HSPCs (red arrowheads) in Tg (*cmyb*:EGFP) embryos was decreased in the AGM region in *142*^T−/−^ embryos at 36 hpf (mean±s.d., *n*=5, *P<0.05). White arrowheads indicate *cmyb*-positive cells in the pronephric duct. *cmyb*-labeled pronephric duct was counted as an internal control. (**e**) *142*^T−/−^ embryos displayed reduction of HSPC marker *cmyb* from 2 to 3.5 dpf by WISH. (**f**) Differentiated hematopoietic lineages were decreased in *142*^T−/−^ embryos including erythrocytes (*gata1*), neutrophils (*lyz*) and lymphocytes (*ikaros* and *rag1*) at 4 dpf by WISH. Red arrowheads mark hematopoietic cells in the caudal hematopoietic tissue (CHT), whereas red circles denote the thymus. (**g**) Hematoxylin and eosin (HE) staining of the kidney showed that decrease in cell population and the volume of the head kidney greatly in *142*^−/−^ adult fish of 12 weeks.

To confirm the HSPC defects in *miR-142a-3p* morphants as reported in our previous study [26], the HSPC marker *runx1* was examined in *142*^T−/−^ embryos and WISH result displayed its reduction in the aorta–gonad–mesonephros (AGM) at 26 and 36 hpf (Figure 2a). In addition, qPCR and western blot analyses also showed similar decrease of *runx1* in *142*^T−/−^ embryos (Figure 2b and c). Consistently, the number of *cmyb*:EGFP^+^ cells (HSPCs) in Tg(*cmyb*:EGFP) embryos was also decreased in *142*^T−/−^ embryos (Figure 2d), whereas *cmyb*-positive cells in the pronephric duct remained unaffected (Figure 2d), demonstrating that the decrease of HSPCs in *142*^T−/−^ embryos is specific. To determine the role of *miR-142a-3p* in the expansion and differentiation of HSPCs at later stages, WISH was performed and the result showed that *cmyb* was decreased in the caudal hematopoietic tissue (the equivalent of fetal liver in mammals) from 2 to 3.5 dpf in *142*^T−/−^ embryos (Figure 2e). Moreover, the differentiated hematopoietic lineages including erythrocytes (labeled by *gata1*), neutrophils (labeled by *lyz*) and T cells (labeled by *ikaros* and *rag1*) were all decreased in *142*^T−/−^ embryos at 4 dpf (Figure 2f), further suggesting that HSPC differentiation was disrupted. Consistent with the results in *142*^T−/−^ embryos, *142*^C−/−^ embryos displayed similar reduction in HSPC markers *runx1* and *cmyb* (Supplementary Figure S3a) and decreased expression of differentiated hematopoietic markers at 4 dpf (Supplementary Figure S3b). Therefore, loss of *miR-142a-3p* leads to the decreased HSPCs and their derivatives in both *142*^T−/−^ and *142*^C−/−^ embryos.

Concerning the absence of *miR-142a-3p* and *miR-142a-5p* in *142*^T−/−^, it is critical to determine whether the decrease in HSPCs is also attributed to the absence of *miR-142a-5p*. In fact, knockdown of *miR-142a-5p* by antisense morpholino showed normal expression of HSPC marker *runx1* (Supplementary Figure S3c), excluding the involvement of *miR-142a-5p* in HSPC regulation. Therefore, we concluded that the defects of HSPCs in *142*^T−/−^ embryos were only attributed to the absence of *miR-142a-3p*.

To demonstrate whether the defect of HSPCs in *142*^T−/−^ can recover during adulthood, the kidney of adult mutant was sectioned and stained with hematoxylin and eosin to test the number of hematopoietic cells. It was observed that both the cellularity and the volume of the pronephros decreased dramatically in 12-week-old *142*^T−/−^ adult fish (Figure 2g), confirming the continuous defect of definitive hematopoiesis in adult zebrafish upon *miR-142a* deletion. To test whether there exists hypermaturation of neutrophils in *142*^T−/−^ as reported previous [24], we also sorted myeloid cells using flow cytometry (FACS) and conducted Wright–Giemsa staining of eosinophils, macrophages and neutrophils. However, our analysis showed that the morphology of myeloid cells in between mutants and WT siblings seems no difference (Supplementary Figure S4), suggesting normal maturation of neutrophils in *142*^T−/−^ embryos.

**Figure 3:**
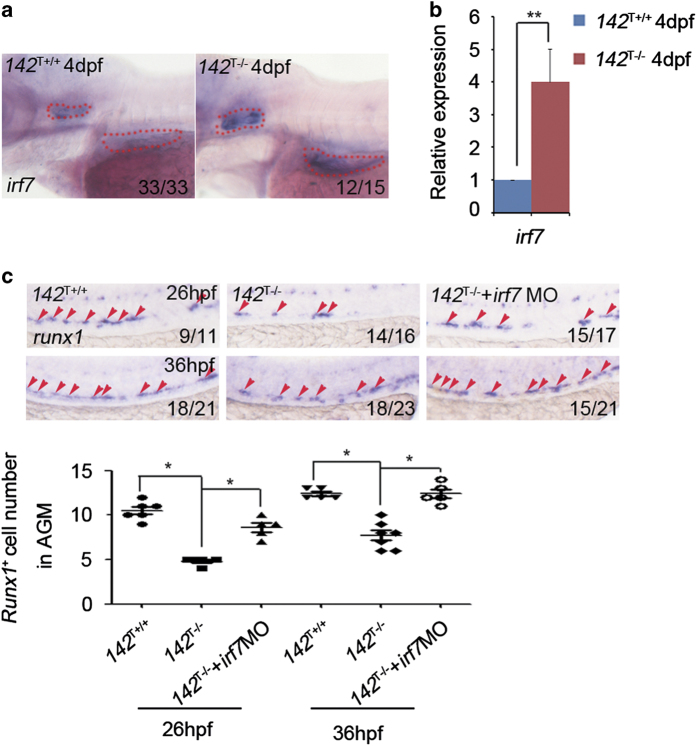
Increased expression of *irf7* in *142*^T−/−^ embryos and rescue of HSPC defects by *irf7* knockdown. (**a** and **b**) *142*^T−/−^ embryos showed increased expression of *irf7* at 4 dpf using WISH and qPCR (mean±s.d., *n*=3, *P<0.01). (**c**) *Irf7* MO-injected *142*^T −/−^ embryos showed the restoration of *runx1* expression at 26 and 36 hpf using WISH. The quantification of *runx1*-positive cells in the AGM was shown in the lower panel (mean±s.d., *n*=5, *P<0.05).

Previous work showed that *irf7* is a direct target of *miR-142a-3p*, and *miR-142a-3p* morphants display increased expression of *irf7* [26]. Using WISH and qPCR, we were able to detect that there was an ectopic expression of *irf7* in *142*^T−/−^ embryos (Figure 3a and b). Moreover, knockdown of *irf7* in *142*^T−/−^ embryos displayed a partial rescue on HSPCs at both 26 and 36 hpf (Figure 3c), confirming that *irf7* functions downstream of *miR-142a-3p* to mediate HSPC development.

Taken together, *miR-142a-3p* is indispensable for the HSPC emergence and differentiation, and its direct target *irf7* is upregulated in *142*^T−/−^ embryos, confirming the findings in our previous study [26].

### Decreased proliferation and increased apoptosis of HSPCs in *142*^T−/−^ embryos

**Figure 4:**
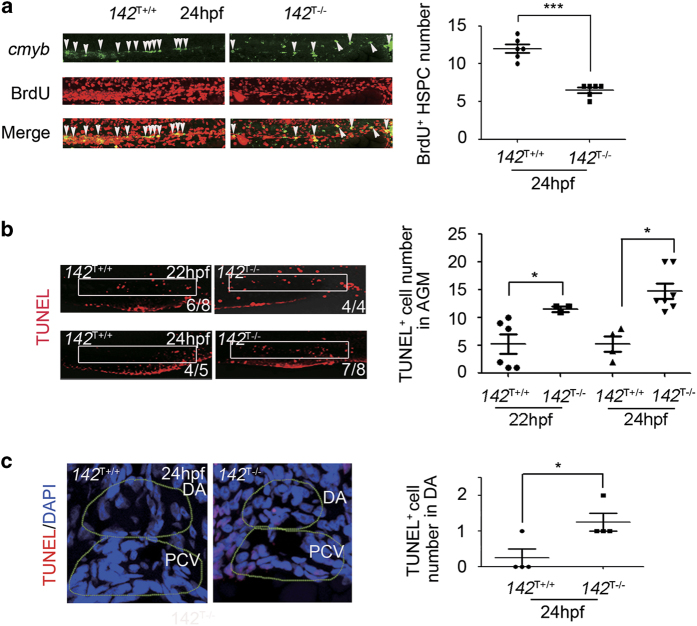
*142*^T−/−^ embryos display decreased proliferation and increased apoptosis of HSPCs. (**a**) Percentage of the proliferative HSPCs labeled by BrdU (marked by white arrow heads) in the AGM of *142*^T−/−^ embryos and wild-type siblings 24 hpf (mean±s.d., *n*=6, *P<0.001). (**b**) TUNEL assay showed more apoptotic cells (white box) in the AGM of *142*^T−/−^ embryos at 22 and 24 hpf. The number of TUNEL-positive cells in the AGM region was quantified (mean±s.d., *n*=3, *P<0.05). (**c**) Transverse section showed increased apoptotic HSPCs in the AGM region of *142*^T−/−^ embryos. Yellow dashed circles denote the dorsal aorta and cardinal vein. TUNEL-positive cells were counted in the dorsal aortal region (mean±s.d., *n*=4, *P<0.05).

To determine how HSPCs were decreased in *142*^T−/−^ embryos, we performed 5-Bromo-2-deoxyuridine (BrdU) labeling for cell proliferation and terminal dexynucleotidyl transferase-mediated dUTP nick end labeling (TUNEL) staining for apoptosis assays. First, we examined HSPC proliferation by counting the proportion of BrdU immunostaining-positive cells in total HSPCs (labeled by *cmyb*-positive cells). The number of proliferative HSPCs (BrdU) was decreased in the AGM in *142*^T−/−^ embryos compared with WT siblings at 24 and 36 hpf, respectively (Figure 4a). These data together indicated that proliferation of emerging HSPCs in the AGM region of *142*^T−/−^ embryos was affected.

Next, we found that *142*^T−/−^ embryos showed increased TUNEL-positive cells in the AGM region at 22 and 24 hpf by TUNEL assay (Figure 4b). Frozen section of the AGM region revealed an increased number of TUNEL-positive cells in the dorsal aorta at 24 hpf (Figure 4c). Consistent with that, *142*^C−/−^ also displayed excessive TUNEL signals in the AGM region at 24 hpf (Supplementary Figure S5a). Therefore, cells in this region including HSPCs underwent excessive apoptosis in both types of *miR-142a* mutants.

On the basis of the decrease in proliferation and the increase in apoptosis of HSPCs in *142*^T−/−^ embryos, we referred to our previous microarray data that contained upregulated genes in *miR-142a-3p* morphants [26] and found that apoptotic genes including *p53* were greatly upregulated (Supplementary Figure S5b). Because of the dual roles of *p53* in both cell cycle arrest and apoptosis and the hint from the microarray, we next focused on the relationship between *miR-142a-3p* and *p53*.

### *142*^T−/−^ embryos display ectopic expression of *p53*

**Figure 5:**
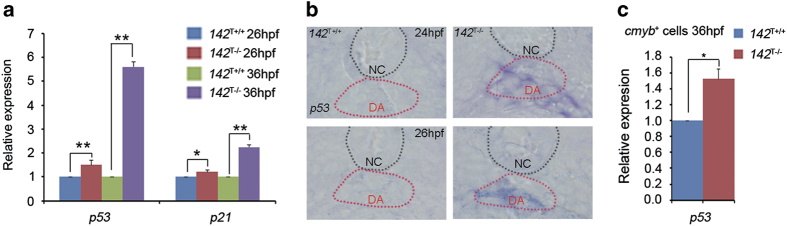
*142*^T−/−^ embryos display ectopic expression of *p53*. (**a**) The expression of *p53* and *p21* was increased in *142*^T−/−^ embryos at 26 and 36 hpf using qPCR (mean±s.d., *n*=3, *P<0.05, **P<0.01). (**b**) *P53* was increased in the dorsal aorta region in *142*^T−/−^ embryos at 24 and 26 hpf by WISH. (**c**) The expression of *p53* was increased in *cmyb*-positive cells in the trunk of *142*^T−/−^ embryos at 36hpf.

To elucidate whether the expression of *p53* in *miR-142a* mutants is consistent with the microarray data in morphants, we first examined the expression of *p53* and its downstream target *p21*. qPCR showed that the expression levels of *p53* and *p21* were increased at 26 and 36 hpf (Figure 5a). To find out whether *p53* is increased in HSPCs, frozen section of *142*^T−/−^ embryos stained with *p53* probe was performed and the result displayed that *p53* was ectopically expressed especially in the dorsal aortal region (Figure 5b). To further support this notion, we sorted *cmyb*-positive cells from the trunk region of *142*^T−/−^ embryos at 36hpf for qPCR analysis. Result showed that expression of *p53* was increased in *cmyb*-positive cells in *142*^T−/−^ (Figure 5c). Taken together, we concluded that *p53* was specifically upregulated in HSPCs of *142*^T−/−^ embryos.

### *MiR-142a-3p* inhibits *p53* by direct binding

On the basis of several studies revealing that *p53* could act as a direct target of microRNAs [41] and the increase of *p53* in *142*^T−/−^ embryos, we sought for the possibility that *p53* might act as a direct target of *miR-142a-3p*. Systematic bioinformatic analysis using RNA hybrid website (BiBiServ2) revealed that there is a *miR-142a-3p*-binding site in the coding sequence (CDS) but not the 3’-UTR of zebrafish *p53*, suggesting that *miR-142a-3p* may be a direct regulator of *p53*.

**Figure 6:**
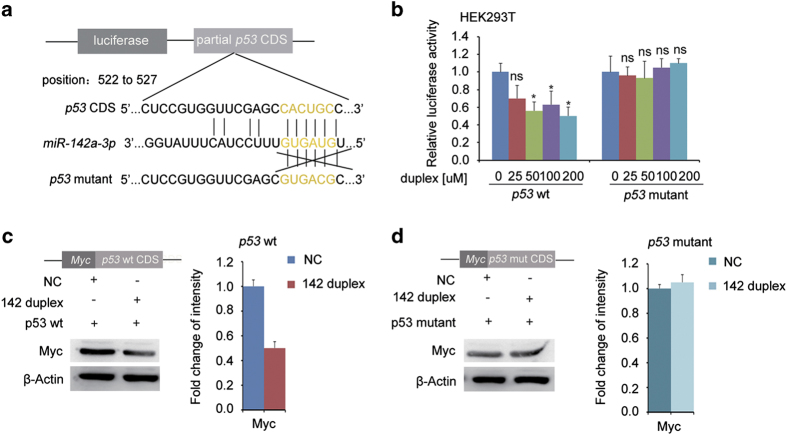
*MiR-142a-3p* binds to the coding region of *p53* mRNA. (**a**) The scheme of the reporter constructs containing the *miR-142a*-binding site and luciferase reporter. The coding region of *p53* was inserted downstream of the luciferase gene. The microRNA-binding sites were predicted by RNA hybrid website. (**b**) Luciferase reporter assay showed that there was a suppression of the luciferase activity in wild-type but not mutated *p53* in a dose-dependent manner in HEK293T cells (mean±s.d., *n*=3, *P<0.05, ns stands for no significance). (**c**) Overexpression of the *miR-142-3p* duplex decreased the protein level of wild-type *p53*. The construct of Myc-tagged wild-type *p53*-coding region (CDS) and western blotting result, left panel; Quantification of western blot, right panel. Myc antibody serves as a tag to evaluate the expression of *p53*. NC stands for negative control of the *miR-142-3p* duplex. (**d**) Overexpression of the *miR-142-3p* duplex had no effect on the protein level of mutated *p53*. The construct of Myc-tagged mutated *p53* CDS and western blot result, left panel; quantification of western blot analysis, right panel.

To confirm the direct targeting of *p53* by *miR-142a-3p,* we cloned the WT *p53*-coding region as well as mutated sequences (mismatched with *miR-142a-3p* seed sequence) containing the predicted *miR-142a-3p*-binding site into luciferase and GFP reporter vector (Figure 6a, Supplementary Figure S6a and b). Luciferase activity and GFP expression were examined, respectively. Overexpression of the *miR-142a-3p* duplex in HEK293T cells and zebrafish embryos suppressed the luciferase activity of WT but not the mutated *p53*-coding region in a dose-dependent manner (Figure 6b, Supplementary Figure S6a), indicating that *p53* is a direct target of *miR-142a-3p*. Moreover, injection of GFP reporter fused with the WT *p53* but not the mutated *p53*-coding region together with the *miR-142a-3p* duplex displayed a marked inhibition of GFP activity (Supplementary Figure S6b). Taken together, these data strongly suggest that *p53* acts as a direct target of *miR-142a-3p*.

To further demonstrate the inhibition of *p53* by *miR-142a-3p* directly, we ectopically expressed the full-length *p53* cDNA fused with *Myc*-tag in the HEK293T cells. The WT or mutated *p53* CDS was co-transfected with the *miR-142a-3p* duplex. Consistent with the reporter assays, overexpression of *miR-142a-3p* could attenuate Myc-P53 expression in WT *p53* but not the mutated *p53*-transfected cells (Figure 6c and d), further supporting the specific binding of *miR-142a-3p* to *p53*.

### *P53* mediates the decrease in HSPCs upon loss of *miR-142a-3p*

**Figure 7:**
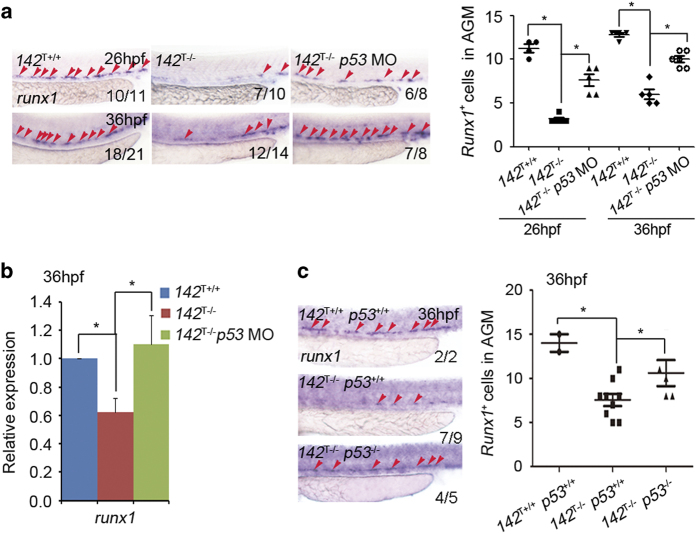
*P53* mediates the decrease of HSPCs in *miR-142a-3p* mutants. (**a**) *P53* MO-injected *142*^T−/−^ embryos showed the restoration of *runx1* expression at 26 and 36 hpf by WISH (left panel). The quantification of *runx1*-positive cells in the AGM was shown in the right panel (mean±s.d., *n*=3, *P<0.05). (**b**) qPCR showed that compared with *142*^T−/−^ at 36 hpf, the decrease in *runx1* expression was rescued in *142*^T −/−^ embryos injected with *p53* MO (mean±s.d., *n*=3, *P<0.05). (**c**) WISH showed the expression of *runx1* in *142*^T+/+^ *p53*^*+/+*^, *142*^T−/−^ *p53*^*+/+*^ *and 142*^T−/−^ *p53*^*−/−*^ embryos at 36 hpf. There was a rescue effect on the decreased *runx1* expression in *142*^T −/−^ *p53*^*−/−*^ double mutants. The quantification of *runx1*-positive cells in the AGM was shown in the right panel (mean±s.d., *n*=60, *P<0.05).

**Figure 8:**
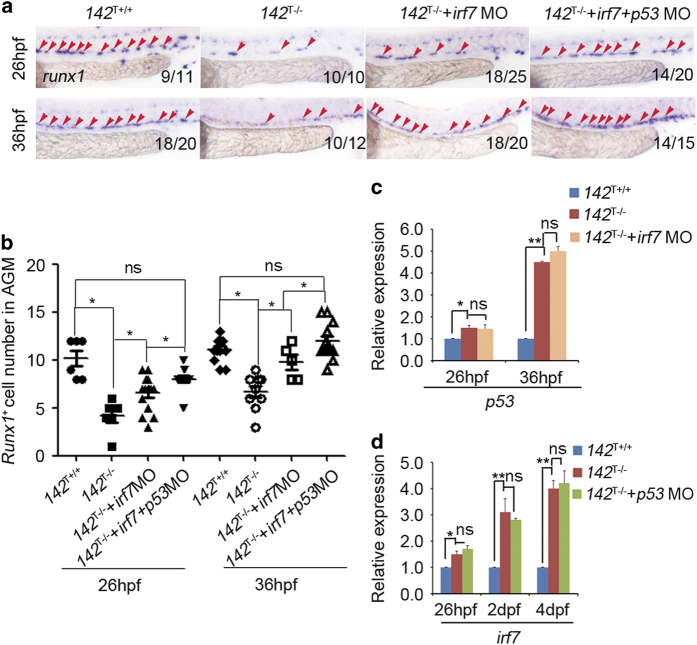
Combined knockdown of *irf7* and *p53* restores decreased HSPCs in *142*^T −/−^ embryos. (**a** and **b**) Injection of *irf7* MO with *p53* MO rescued the decreased expression of HSPCs marker *runx1*, and the extent of the restoration was greater than the single injection of *irf7* MO at 26 and 36 hpf (mean±s.d., *n*=5, *P<0.05). (**c**) The increased expression of *p53* was not affected in *142*^T−/−^ embryos injected with *irf7* MO at 26 and 36 hpf by qPCR (each sample was composed at least 40 embryos, mean±s.d., *n*=3, *P<0.05, **P<0.01, ns stands for no significance). (**d**) The increased expression of *irf7* was not affected in *142*^T−/−^ embryos injected with *p53* MO at 26 hpf, 2 and 4 dpf by qPCR (mean±s.d., *n*=3, *P<0.05, **P<0.01, ns stands for no significance).

To determine whether *p53* upregulation mediated the HSPC defects in *142*^T−/−^ embryos, we first injected *p53* morpholino (MO) into *142*^T−/−^ embryos. WISH and qPCR results showed that *p53* MO could partially rescue the decreased expression of *runx1* in *142*^T−/−^ embryos at 26 and 36 hpf (Figure 7a and b). Furthermore, we generated a double knockout of *miR-142a-3p* and *p53* by outcrossing adult *142*^T−/−^ with *p53*^M214K−/−^. WISH showed that the expression of *runx1* at 36 hpf was markedly restored in *142*^T−/−^ *p53*^M214K−/−^ embryos, compared with *142*^T−/−^ *p53*^M214K+/+^ embryos (Figure 7c), consistent with the results by *p53* MO knockdown. To determine whether there is a synergy effect between *irf7* and *p53* in *miR-142a-3p*-mediated HSPC development, we knocked down both *irf7* and *p53* in *142*^T−/−^ embryos. Double knockdown of *irf7* and *p53* fully rescued the HSPC defect (Figure 8a and b), demonstrating that *irf7* and *p53* act synergistically downstream of *miR-142a-3p* in HSPC development. In addition, the expression of *p53* and *irf7* was examined in *miR-142a* mutants injected with irf7 MO or p53 MO, respectively. qPCR showed that knockdown of *irf7* or *p53* had no effect on the expression of each other (Figure 8c and d), revealing that *irf7* and *p53* are parallel targets of *miR-142a-3p*.

In order to test that *p53* inhibition specifically restored apoptosis in the AGM region, we injected *p53* MO into *142*^T−/−^ embryos. As expected, *p53* MO injection led to alleviated apoptosis in both the trunk and AGM region of *142*^T−/−^ embryos (Supplementary Figure S7a), strengthening the cause–consequence relationship between *p53*-induced apoptosis and HSPC defects in *miR-142a-3p* mutant.

In addition, we also examined the expression of differentiated hematopoietic markers in *p53* MO-injected *142*^T−/−^ mutants. As shown in Figure S7b, the defects of differentiated populations including erythrocytes, neutrophils and T cells in *142*^T−/−^ embryos were also partially restored by *p53* knockdown, supporting that *p53* functions downstream of *miR-142a-3p* in HSPC differentiation.

## Discussion

In this work, by generating two types of *miR-142a-3p* mutants using both TALENs and CRISPR/Cas9 methods, we demonstrate that *miR-142a-3p* has an essential role in definitive hematopoiesis. Decreased HSPCs and their derivatives, as well as ectopic expression of *p53* and *irf7*, were observed in *miR-142a-3p* mutants. Loss of *p53* could rescue the defects of HSPCs in *miR-142a-3p* mutants and direct binding assay together support that *p53* is a direct target of *miR-142a-3p*, and the newly identified *miR-142a-3p-p53* axis has a critical role in HSPC survival and thus protects emerging HSPCs and their derivatives from apoptosis. In addition, *p53* acts synergistically with the previously reported *irf7* downstream of *miR-142a-3p* in controlling HSPC development [26] (Supplementary Figure S8).

Consistent with our previous finding that *miR-142a-3p* is highly expressed in the *runx1*^*+*^/*cmyb*^*+*^ cells in the trunk region that labels HSPC populations in zebrafish [26], two independent groups also showed evidence that the counterpart of *miR-142a-3p* is enriched in HSPC population in Xenopus and mice [22, 23]. Studies in Xenopus demonstrated that *miR-142a-3p* targets *TGFβ* and functions as a master regulator in HSPC lineage specification and thus sits at the apex of the hierarchy programming definitive hemangioblast [23]. This is consistent with our work and illustrates the crucial function of *miR-142a-3p* in definitive hematopoiesis across vertebrates.

However, there are also different views on the function of *miR-142a-3p* in HSPCs based on morpholino or duplex injection [27, 40]. It was reported that ectopic expression of the *miR-142-3p* duplex (concentration of 20 μmol l^−1^) suppresses the primitive erythrocyte progenitors (*gata1*^*+*^) and HSC formation (*cmyb*^*+*^) [27]. We also performed *miR-142a-3p* duplex injection and found that the dose of duplex they used caused nonspecific side effects in embryos, such as heart edema, whereas lower dose (concentration of 5–0 μmol l^−1^) showed increased expression of HSPC marker *runx1* at 26 hpf as well as *cmyb* and *rag1* at 5 dpf. To exclude the delusive dose effect of morpholino or duplex, in this work, we chose to generate *miR-142a* genetic mutants for functional investigation.

Recently, by targeting two *miR-142* genes including *miR-142a* and *miR-142b,* the mutants of *miR-142* by zinc-finger nucleases (ZFN) method are generated, and these *miR-142-3p* mutants display neutrophil defects but with relatively normal HSPCs [24]. Consistently, our *142*^T−/−^ embryos also displayed reduced number of neutrophils (marked by *lyz*) by WISH at 4 dpf (Figure 2f). Furthermore, the kidney, which contains more neutrophils compared with other organs [42], was shrunken in *142*^T−/−^ adult fish (Figure 2g). Notably, hematoxylin and eosin staining result revealed that the cellular density of neutrophils was decreased during the loss of *miR-142*. According to the Giemsa staining, the maturation of neutrophil was normal, as different subtypes of neutrophils with various morphologies were all observed (Supplementary Figure S4). This result is discrepant with the previously reported neutrophil phenotypes in *miR-142a/b* mutants [24]. On the basis of the deficiency of miR*-142a* and *miR-142b* genes, there is a possibility that the reported neutrophil hypermaturation in *miR-142a/b* mutants might be due to the deletion of *miR-142b*. Moreover, neutrophils at different maturation stages can be observed in WT and *142*^T−/−^ embryos; therefore, the ratio of naive versus mature neutrophils varies with development.

As a tumor suppressor, *p53* is also a critical transcription factor in hematopoietic cells and it is involved in the quiescence, self-renewal, senescence and apoptosis of HSPCs [32, 33]. It also modulates the cell cycle arrest of HSPCs through *p21* [32]. Notably, mounting evidence suggests that HSPCs are highly sensitive to cellular stress, cell cycling and the microenvironment, which are closely related to the function of *p53* [43, 44]. In zebrafish, mutation in cleavage and polyadenylation specificity factor 1 (*cpsf1*) displays definitive HSC defects due to a *p53*-dependent apoptotic cell death [45]. In mouse, the depletion of HSPCs in mutants of *Mysm1* and *Pot1b* could be restored by loss of the *P53* [38, 46]. These findings support that HSPC survival is tightly controlled by various factors and that *p53*-mediated apoptosis is the key to maintain the survival of HSPCs. Our work here demonstrates that in zebrafish embryogenesis, the activation of P53 in the AGM region leads to excessive apoptosis, which impairs the survival of the earliest specified HSPCs. Consistent with our findings, super-*p53* mice, carrying one extra gene dose of *p53*, results in significant acquisition of repopulating capacity but decreased survival of HSCs in recipients [47]. Direct and indirect evidence sheds light upon the hypothesis that the dosage of *p53* should be strictly controlled to maintain the normal HSPC survival. Although several studies have identified the downstream targets of *p53* in regulating HSPCs, the direct upstream regulators of *p53* in modulating definitive hematopoiesis are not well defined. Here, we show that *miR-142a-3p* directly binds to the coding region of *p53* and blocks its function, and then reveals the crucial role of the *miR-142a-3p*-*p53* axis in HSPC survival.

In our previous study, *irf7* knockdown only partially rescued the HSPC defects in *miR-142a-3p* morphants [26], suggesting that other targets of *miR-142a-3p* may be involved in this process. In fact, double knockdown of *irf7* and *p53* could fully rescued the HSPC defects and the rescue effect was better compared with *irf7* knockdown, demonstrating that there is a synergistic effect of both *irf7* and *p53* downstream of *miR-142a-3p* in HSPC development. Tight control of HSPC survival is crucial for HSPC development at early stages in vertebrates. It has been reported that *scl-a*-deficient zebrafish embryos display more apoptosis and failure to maintain the survival of nascent HSPCs in the AGM [48]. In mice, knockout of *gata2* in hematopoietic cells using *Vav*-Cre shows increased apoptosis and attenuated survival of HSPCs [49]. Interestingly, a recent report also showed that *miR-142*^−/−^ CD4^+^ DCs underwent excessive apoptosis, compared with their littermate counterparts [50]. Although developmental defects in HSPCs were not examined in *miR-142*^−/−^ mice, we speculate that a similar HSPC phenotype would be expected at the onset of definitive hematopoiesis. Recently, inhibition of *p53* and its downstream targets *p21* or *miR-34a* has been demonstrated to enhance the reprogramming efficiency of induced pluripotent stem (iPS) cells generation [51, 52]. Moreover, repression of *p53* can efficiently induce CD34^+^ cord blood cells to iPSCs [53]. These lines of evidence in stem cells give clues that excessive level of *p53* might dampen the emergence of stem cells and *miR-142a-3p* could protect the nascent emerging HSPCs from unwanted apoptosis, and this protection is critical for HSPC development during embryogenesis.

In summary, our studies demonstrate that *miR-142a-3p* directly targets *p53* to regulate HSPC survival in zebrafish embryos. In addition to our previous report in which *irf7* is a direct target of *miR-142a-3p* in controlling HSPC formation and differentiation, here we discover that *p53* acts downstream of *miR-142a-3p* to control the survival of HSPCs.

## Materials and Methods

### Generation of *miR-142a* mutants and RNA secondary structure prediction

Zebrafish *miR-142a* mutants were generated by TALEN and CRISPR/Cas9, as previously described [54, 55]. The injection dose of *miR-142a* TALEN mRNA was 250 pg per embryo. The injection doses of gRNA and *cas9* mRNA were 100 and 500 pg per embryo, respectively. The TALEN-generated *miR-142a* mutants (*142*^T−/−^) had a 13-base-pair deletion and could be verified by sequencing (Supplementary Table S1). The CRISPR/Cas9-generated *miR-142a* mutants (*142*^C−/−^) caused a 986-base-pair deletion, and the structure of mature *miR-142a-3p* in both mutants was disrupted. The *142*^C−/−^ could be genotyped by a combination of different lengths of PCR products (Supplementary Table S2). The microRNA secondary structure was predicted by the RNA Structure software (version 4.6).

### Fish strains and embryos

Zebrafish strains including WT Tubingen, *142*^T−/−^, *142*^C−/−^, *p53*^M214K^, *cmyb*:EGFP/*142*^T+/−^ and *142*^T+/−^ *p53*^M214K+/−^ were raised and maintained in system water at 28.5  °C. Fish line *p53*^M214K^ [56] was kindly provided by Dr Anming Meng (Tsinghua University). This study was approved by the Ethical Review Committee in the Institute of Zoology, Chinese Academy of Sciences, China.

### Morpholinos and microinjection

Antisense morpholinos were ordered from Gene Tools including *p53* MO and *irf7* MO. The *p53* MO: 5′-GCGCCATTGCTTTGCAAGAATTG-3′ was injected at the dose of 4 ng per embryo and the *irf7* MO was conducted as previously described [26].

### WISH

Antisense RNA probes were synthesized with SP6 or T7 RNA polymerase (Promega, Madison, WI, USA). WISH for *miR-142a-3p* was conducted at 52 °C and for others were performed at 65 °C with probes including *miR-142a-3p*, *scl*, *pu.1*, *msr*, *flt4*, *dll4*, *dltC*, *efnB2a*, *runx1*, *cmyb*, *gata1, lyz, ikaros, rag1* and *p53*. Numbers in the bottom right denote the ratio of representative embryos in the total scored embryos. At least three independent experiments were conducted for each analysis.

### Quantitative RT-PCR and western blot analyses

Trunk regions of embryos were dissected for qPCR and western blot analyses. Quantitative RT-PCR and western blot analyses were performed as described previously [57]. Antibody used for western blot analysis included anti-Runx1(1:200, AnaSpec, Fermont, CA, USA) and anti-Myc (1:500, Cell Signaling, Danvers, MA, USA). Anti-Myc antibody was used as a tag to test the binding affinity of *miR-142a-3p* to *p53*.

### Reporter assay

The WT and mutated *p53*-coding regions were cloned and then inserted into the *Eco*RI and *Spe*I sites of the pGL3-luciferase vector, which was kindly provided by Dr Peifeng Li (Institute of Zoology, CAS, Beijing, China). The WT p53-coding region was mutated from ‘CACUGC’ to ‘GUGAUG’. Wild-type or mutated pGL3-*p53* CDS co-transfected with negative control or the *miR-142-3p* duplex (Invitrogen, Shanghai, China) were transfected into HEK293T cells using Lipofectamine2000 (Invitrogen). The luciferase activity was measured using the luciferase activity assay kit (Promega). Luciferase and GFP reporter assay in zebrafish was conducted as described previously [24, 58].

### Immunostaining, BrdU incorporation and TUNEL assay and section

Live zebrafish embryos injected with BrdU were fixed in 4% paraformaldehyde (PFA) overnight and then replaced with methanol at −20 °C for 2 hpf. Embryos were permeabilized with proteinase K, refixed in 4% PFA for 20 min and then rinsed in PBST, followed by incubation in 2 n HCl for 1 h. Then, embryos were blocked for 1 h in blocking solution and incubated with primary anti-BrdU antibody (Roche, Mannheim, Germany) and secondary Alexa Fluor 488-conjugated anti-mouse antibody (Invitrogen). The BrdU-labeled embryos were treated with 30% sucrose and then washed three times using PBST and embedded in O.C.T. medium (SAKURA, Torrance, CA, USA). The embryos were sectioned using LEICACM1900 cryostats. Detection of both *cmyb* RNA and mitosis marker pH3 and BrdU simultaneously was conducted as described previously [59].

Embryos at 24 hpf in methanol were washed with PBST and then permeabilized with proteinase K. After being washed with PBST, embryos were subjected to TUNEL assay according to the instruction of the TUNEL staining kit (Roche) at 4 °C overnight.

### Flow cytometry of transgenic fish and whole-kidney marrow

Flow cytometry analysis and cell sorting were based on the forward scatter and side scatter as described previously [42]. All experiments were conducted using the FACSCalibur flow cytometer (BD, San Jose, CA, USA). Data was analyzed using the FlowJo software Version 7.6.1.

### Hematoxylin and eosin and Wright–Giemsa

Hematoxylin and eosin staining was performed on the serial sections of larval zebrafish. Wright–Giemsa staining was performed as described previously [42].

### Microscopy

Embryos were observed using a Nikon microscope for digital image capture. Confocal images of live *cmyb*:EGFP embryos, TUNEL and BrdU assays were taken on confocal microscope (Nikon A1 laser microscope, Nikon A1, Melville, NY, USA). All images were assembled using Photoshop (Adobe Systems, Mountain View, CA, USA).

### Statistical analysis

Values are presented as mean±s.d. Student’s *t*-test was used to compare the means of different groups and *P*-values<0.05 were considered to be significant (*P<0.05, **P<0.01).

## References

[bib1] Wang LD , Wagers AJ . Dynamic niches in the origination and differentiation of haematopoietic stem cells. Nat Rev Mol Cell Biol 2011; 12: 643–655. 21886187 10.1038/nrm3184 PMC4040463

[bib2] Bertrand JY , Chi NC , Santoso B , Teng S , Stainier DY , Traver D . Haematopoietic stem cells derive directly from aortic endothelium during development. Nature 2010; 464: 108–111. 20154733 10.1038/nature08738 PMC2858358

[bib3] Boisset JC , van Cappellen W , Andrieu-Soler C , Galjart N , Dzierzak E , Robin C . *In vivo *imaging of haematopoietic cells emerging from the mouse aortic endothelium. Nature 2010; 464: 116–120. 20154729 10.1038/nature08764

[bib4] Jaffredo T , Gautier R , Eichmann A , Dieterlen-Lievre F . Intraaortic hemopoietic cells are derived from endothelial cells during ontogeny. Development 1998; 125: 4575–4583. 9778515 10.1242/dev.125.22.4575

[bib5] Jaffredo T , Bollerot K , Sugiyama D , Gautier R , Drevon C . Tracing the hemangioblast during embryogenesis: developmental relationships between endothelial and hematopoietic cells. Int J Dev Biol 2005; 49: 269–277. 15906241 10.1387/ijdb.041948tj

[bib6] Clements WK , Kim AD , Ong KG , Moore JC , Lawson ND , Traver D . A somitic Wnt16/Notch pathway specifies haematopoietic stem cells. Nature 2011; 474: 220–262. 21654806 10.1038/nature10107 PMC3304471

[bib7] Espin-Palazon R , Stachura DL , Campbell CA et al. Proinflammatory signaling regulates hematopoietic stem cell emergence. Cell 2014; 159: 1070–1085. 25416946 10.1016/j.cell.2014.10.031 PMC4243083

[bib8] Kim AD , Melick CH , Clements WK et al. Discrete Notch signaling requirements in the specification of hematopoietic stem cells. EMBO J 2014; 33: 2363–2373. 25230933 10.15252/embj.201488784 PMC4253525

[bib9] Kobayashi I , Kobayashi-Sun J , Kim AD et al. Jam1a-Jam2a interactions regulate haematopoietic stem cell fate through Notch signalling. Nature 2014; 512: 319–323. 25119047 10.1038/nature13623 PMC4237229

[bib10] Wilkinson RN , Pouget C , Gering M et al. Hedgehog and Bmp polarize hematopoietic stem cell emergence in the zebrafish dorsal aorta. Dev Cell 2009; 16: 909–916. 19531361 10.1016/j.devcel.2009.04.014 PMC3210643

[bib11] Bresciani E , Carrington B , Wincovitch S et al. CBF beta and RUNX1 are required at 2 different steps during the development of hematopoietic stem cells in zebrafish. Blood 2014; 124: 70–78. 24850758 10.1182/blood-2013-10-531988 PMC4125354

[bib12] de Pater E , Kaimakis P , Vink CS et al. Gata2 is required for HSC generation and survival. J Exp Med 2013; 210: 2843–2850. 24297996 10.1084/jem.20130751 PMC3865477

[bib13] Ren X , Gomez GA , Zhang B , Lin S . Scl isoforms act downstream of etsrp to specify angioblasts and definitive hematopoietic stem cells. Blood 2010; 115: 5338–5346. 20185582 10.1182/blood-2009-09-244640 PMC2902133

[bib14] Solaimani Kartalaei P , Yamada-Inagawa T , Vink CS et al. Whole-transcriptome analysis of endothelial to hematopoietic stem cell transition reveals a requirement for Gpr56 in HSC generation. J Exp Med 2014; 212: 93–106. 25547674 10.1084/jem.20140767 PMC4291529

[bib15] Trowbridge JJ , Snow JW , Kim J , Orkin SH . DNA methyltransferase 1 is essential for and uniquely regulates hematopoietic stem and progenitor cells. Cell Stem Cell 2009; 5: 442–449. 19796624 10.1016/j.stem.2009.08.016 PMC2767228

[bib16] Luo M , Jeong M , Sun D et al. Long non-coding RNAs control hematopoietic stem cell function. Cell Stem Cell 2015; 16: 426–438. 25772072 10.1016/j.stem.2015.02.002 PMC4388783

[bib17] Chen CZ , Li L , Lodish HF , Bartel DP . MicroRNAs modulate hematopoietic lineage differentiation. Science 2004; 303: 83–86. 14657504 10.1126/science.1091903

[bib18] Liu J , Li W , Wang S et al. MiR-142-3p attenuates the migration of CD4(+) T cells through regulating actin cytoskeleton via RAC1 and ROCK2 in arteriosclerosis obliterans. PLoS ONE 2014; 9: e95514. 24743945 10.1371/journal.pone.0095514 PMC3990671

[bib19] Lagrange B , Martin RZ , Droin N et al. A role for miR-142-3p in colony-stimulating factor 1-induced monocyte differentiation into macrophages. Biochim Biophys Acta 2013; 1833: 1936–1946. 23602969 10.1016/j.bbamcr.2013.04.007

[bib20] Kramer NJ , Wang WL , Reyes EY et al. Altered lymphopoiesis and immunodeficiency in miR-142 null mice. Blood 2015; 125: 3720–3730. 25931583 10.1182/blood-2014-10-603951

[bib21] Guo S , Lu J , Schlanger R et al. MicroRNA miR-125a controls hematopoietic stem cell number. Proc Natl Acad Sci USA 2010; 107: 14229–14234. 20616003 10.1073/pnas.0913574107 PMC2922532

[bib22] Pereira CF , Chang B , Qiu JJ et al. Induction of a hemogenic program in mouse fibroblasts. Cell Stem Cell 2013; 13: 205–218. 23770078 10.1016/j.stem.2013.05.024 PMC3735774

[bib23] Nimmo R , Ciau-Uitz A , Ruiz-Herguido C et al. MiR-142-3p controls the specification of definitive hemangioblasts during ontogeny. Dev Cell 2013; 26: 237–249. 23911199 10.1016/j.devcel.2013.06.023

[bib24] Fan HB , Liu YJ , Wang L et al. miR-142-3p acts as an essential modulator of neutrophil development in zebrafish. Blood 2014; 124: 1320–1330. 24990885 10.1182/blood-2013-12-545012

[bib25] Chapnik E , Rivkin N , Mildner A et al. miR-142 orchestrates a network of actin cytoskeleton regulators during megakaryopoiesis. Elife 2014; 3: e01964. 24859754 10.7554/eLife.01964 PMC4067751

[bib26] Lu X , Li X , He Q et al. miR-142-3p regulates the formation and differentiation of hematopoietic stem cells in vertebrates. Cell Res 2013; 23: 1356–1368. 24165894 10.1038/cr.2013.145 PMC3847575

[bib27] Song BF , Zhang Q , Zhang ZJ et al. Systematic transcriptome analysis of the zebrafish model of diamond-blackfan anemia induced by RPS24 deficiency. BMC Genomics 2014; 15: 759. 25189322 10.1186/1471-2164-15-759 PMC4169864

[bib28] Krug U , Ganser A , Koeffler HP . Tumor suppressor genes in normal and malignant hematopoiesis. Oncogene 2002; 21: 3475–3495. 12032783 10.1038/sj.onc.1205322

[bib29] Speidel D . The role of DNA damage responses in p53 biology. Arch Toxicol 2015; 89: 501–517. 25618545 10.1007/s00204-015-1459-z

[bib30] Lowe SW , Schmitt EM , Smith SW , Osborne BA , Jacks T . p53 is required for radiation-induced apoptosis in mouse thymocytes. Nature 1993; 362: 847–849. 8479522 10.1038/362847a0

[bib31] Kuerbitz SJ , Plunkett BS , Walsh WV , Kastan MB . Wild-type p53 is a cell cycle checkpoint determinant following irradiation. Proc Natl Acad Sci USA 1992; 89: 7491–7495. 1323840 10.1073/pnas.89.16.7491 PMC49736

[bib32] Pant V , Quintas-Cardama A , Lozano G . The p53 pathway in hematopoiesis: lessons from mouse models, implications for humans. Blood 2012; 120: 5118–5127. 23018641 10.1182/blood-2012-05-356014 PMC3537308

[bib33] Nii T , Marumoto T , Tani K . Roles of p53 in various biological aspects of hematopoietic stem cells. J Biomed Biotechnol 2012; 2012: 903435. 22778557 10.1155/2012/903435 PMC3388322

[bib34] Dumble M , Moore L , Chambers SM et al. The impact of altered p53 dosage on hematopoietic stem cell dynamics during aging. Blood 2007; 109: 1736–1742. 17032926 10.1182/blood-2006-03-010413 PMC1794064

[bib35] Tyner SD , Venkatachalam S , Choi J et al. p53 mutant mice that display early ageing-associated phenotypes. Nature 2002; 415: 45–53. 11780111 10.1038/415045a

[bib36] Bibikova E , Youn MY , Danilova N et al. TNF-mediated inflammation represses GATA1 and activates p38 MAP kinase in RPS19-deficient hematopoietic progenitors. Blood 2014; 124: 3791–3798. 25270909 10.1182/blood-2014-06-584656 PMC4263986

[bib37] Batta K , Florkowska M , Kouskoff V , Lacaud G . Direct reprogramming of murine fibroblasts to hematopoietic progenitor cells. Cell Rep 2014; 9: 1871–1884. 25466247 10.1016/j.celrep.2014.11.002 PMC4542300

[bib38] Belle JI , Langlais D , Petrov JC et al. p53 mediates loss of hematopoietic stem cell function and lymphopenia in Mysm1 deficiency. Blood 2015; 125: 2344–2348. 25710881 10.1182/blood-2014-05-574111

[bib39] Lalwani MK , Sharma M , Singh AR et al. Reverse genetics screen in zebrafish identifies a role of miR-142a-3p in vascular development and integrity. PLoS ONE 2012; 7: e52588. 23285103 10.1371/journal.pone.0052588 PMC3528674

[bib40] Nishiyama T , Kaneda R , Ono T et al. miR-142-3p is essential for hematopoiesis and affects cardiac cell fate in zebrafish. Biochem Biophys Res Commun 2012; 425: 755–761. 22884798 10.1016/j.bbrc.2012.07.148

[bib41] Le MT , Teh C , Shyh-Chang N et al. MicroRNA-125b is a novel negative regulator of p53. Genes Dev 2009; 23: 862–876. 19293287 10.1101/gad.1767609 PMC2666337

[bib42] Traver D , Paw BH , Poss KD , Penberthy WT , Lin S , Zon LI . Transplantation and *in vivo* imaging of multilineage engraftment in zebrafish bloodless mutants. Nat Immunol 2003; 4: 1238–1246. 14608381 10.1038/ni1007

[bib43] Mohrin M , Shin J , Liu Y et al. Stem cell aging. A mitochondrial UPR-mediated metabolic checkpoint regulates hematopoietic stem cell aging. Science 2015; 347: 1374–1377. 25792330 10.1126/science.aaa2361 PMC4447312

[bib44] Zhang M , Zhu X , Zhang Y et al. RCAD/Ufl1, a Ufm1 E3 ligase, is essential for hematopoietic stem cell function and murine hematopoiesis. Cell Death Differ (e-pub ahead of print 8 May 2015; doi:10.1038/cdd.2015.51). 10.1038/cdd.2015.51 PMC4816109 25952549

[bib45] Bolli N , Payne EM , Rhodes J et al. cpsf1 is required for definitive HSC survival in zebrafish. Blood 2011; 117: 3996–4007. 21330472 10.1182/blood-2010-08-304030 PMC3087528

[bib46] Wang Y , Shen MF , Chang S . Essential roles for Pot1b in HSC self-renewal and survival. Blood 2011; 118: 6068–6077. 21948176 10.1182/blood-2011-06-361527 PMC3234665

[bib47] Herrera-Merchan A , Cerrato C , Luengo G et al. miR-33-mediated downregulation of p53 controls hematopoietic stem cell self-renewal. Cell Cycle 2010; 9: 3277–3285. 20703086 10.4161/cc.9.16.12598

[bib48] Zhen FH , Lan YH , Yan B , Zhang WQ , Wen ZL . Hemogenic endothelium specification and hematopoietic stem cell maintenance employ distinct Scl isoforms. Development 2013; 140: 3977–3985. 24046317 10.1242/dev.097071

[bib49] de Pater E , Kaimakis P , Vink CS et al. Gata2 is required for HSC generation and survival. J Exp Med 2013; 210: 2843–2850. 24297996 10.1084/jem.20130751 PMC3865477

[bib50] Mildner A , Chapnik E , Manor O et al. Mononuclear phagocyte miRNome analysis identifies miR-142 as critical regulator of murine dendritic cell homeostasis. Blood 2013; 121: 1016–1027. 23212522 10.1182/blood-2012-07-445999

[bib51] Hong H , Takahashi K , Ichisaka T et al. Suppression of induced pluripotent stem cell generation by the p53-p21 pathway. Nature 2009; 460: 1132–1135. 19668191 10.1038/nature08235 PMC2917235

[bib52] Choi YJ , Lin CP , Ho JJ et al. miR-34 miRNAs provide a barrier for somatic cell reprogramming. Nat Cell Biol 2011; 13: 1353–1360. 22020437 10.1038/ncb2366 PMC3541684

[bib53] Takenaka C , Nishishita N , Takada N , Jakt LM , Kawamata S . Effective generation of iPS cells from CD34(+) cord blood cells by inhibition of p53. Exp Hematol 2010; 38: 154–162. 19922768 10.1016/j.exphem.2009.11.003

[bib54] Cade L , Reyon D , Hwang WY et al. Highly efficient generation of heritable zebrafish gene mutations using homo- and heterodimeric TALENs. Nucleic Acids Res 2012; 40: 8001–8010. 22684503 10.1093/nar/gks518 PMC3439908

[bib55] Chang N , Sun C , Gao L et al. Genome editing with RNA-guided Cas9 nuclease in zebrafish embryos. Cell Res 2013; 23: 465–472. 23528705 10.1038/cr.2013.45 PMC3616424

[bib56] Berghmans S , Murphey RD , Wienholds E et al. tp53 mutant zebrafish develop malignant peripheral nerve sheath tumors. Proc Natl Acad Sci USA 2005; 102: 407–412. 15630097 10.1073/pnas.0406252102 PMC544293

[bib57] Wei YL , Ma DY , Gao Y , Zhang CX , Wang L , Liu F . Ncor2 is required for hematopoietic stem cell emergence by inhibiting Fos signaling in zebrafish. Blood 2014; 124: 1578–1585. 25006126 10.1182/blood-2013-11-541391

[bib58] Alcaraz-Perez F , Mulero V , Cayuela ML . Application of the dual-luciferase reporter assay to the analysis of promoter activity in Zebrafish embryos. BMC Biotechnol 2008; 8: 81. 18954456 10.1186/1472-6750-8-81 PMC2596795

[bib59] Jia XE , Ma K , Xu T et al. Mutation of kri1l causes definitive hematopoiesis failure via PERK-dependent excessive autophagy induction. Cell Res 2015; 25: 946–962. 26138676 10.1038/cr.2015.81 PMC4528055

