# Supplementary material for: Direct regulation of p53 by miR-142a-3p mediates the survival of hematopoietic stem and progenitor cells in zebrafish
Source: Cell Discov. 2015 Sep 15;1:15027–. doi: 10.1038/celldisc.2015.27 (PMC4860776; doi:10.1038/celldisc.2015.27)
Supplement: Supplementary Information [file celldisc201527-s1.pdf]

Supplementary Figure S1

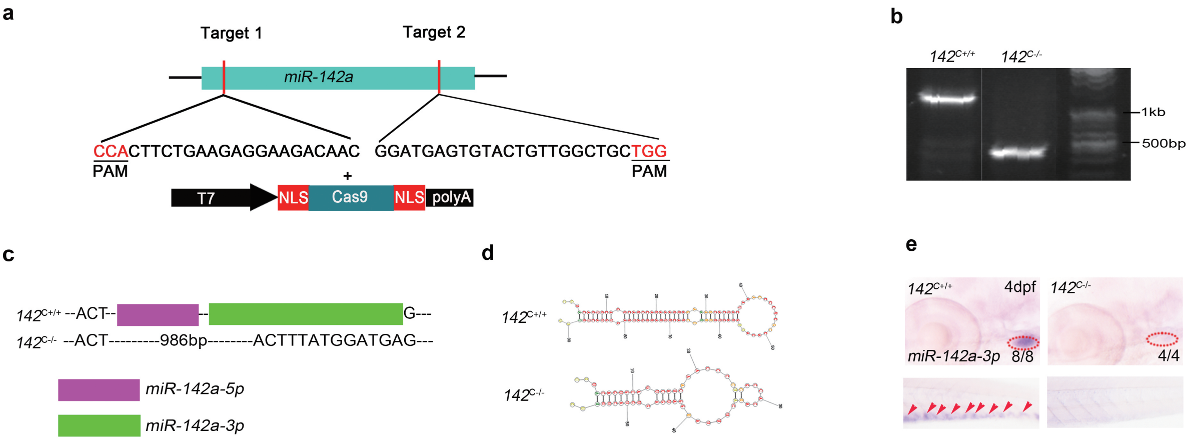

Supplementary Figure S2

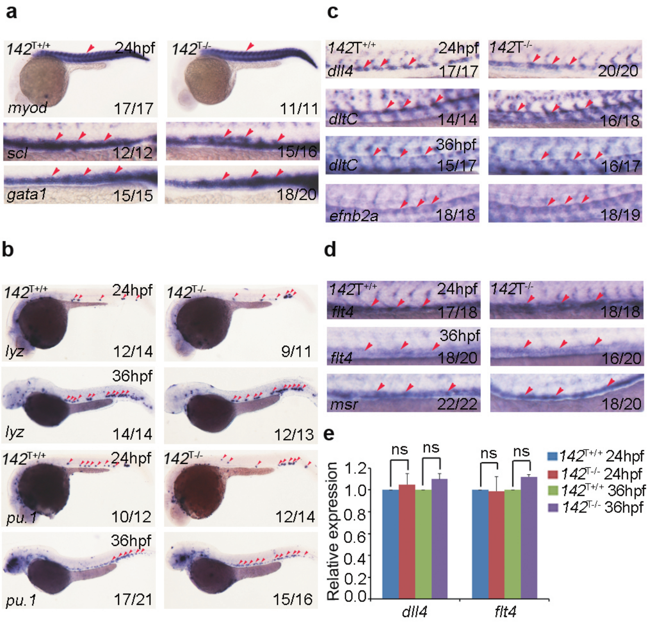

Supplementary Figure S3

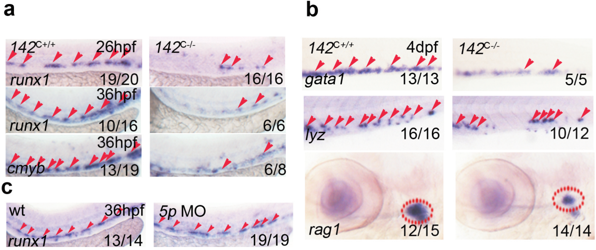

Supplementary Figure S4

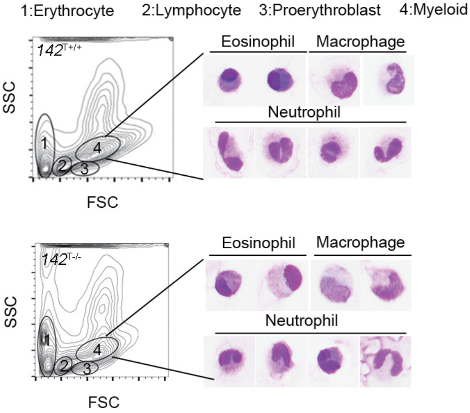

Supplementary Figure S5

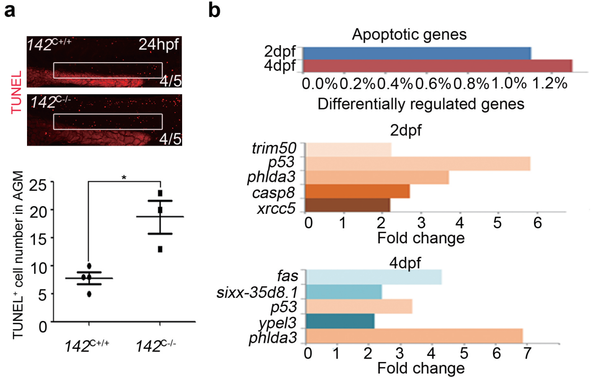

Supplementary Figure S6

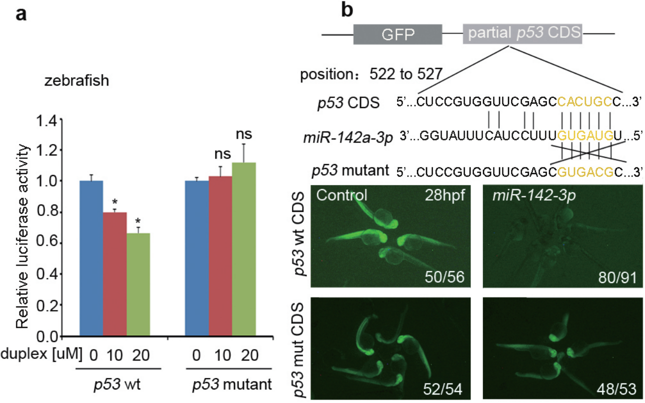

Supplementary Figure S7

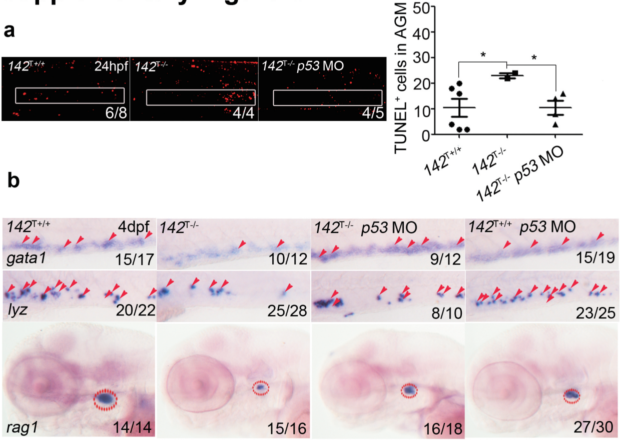

Supplementary Figure S8

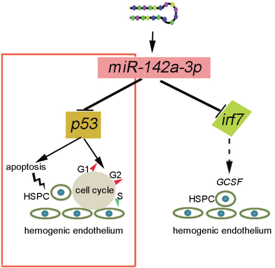

Supplementary figure legends

Supplementary Figure S1. Generation of *miR-142a* knock out zebrafish using CRISPR/Cas9.

(a) Schematic diagram showing the CRISPR/Cas9 target sites 1 and 2 in the *miR-142a* gene. (b) Genotyping showed different lengths of PCR products from *142*$^{C+/+}$ and *142*$^{C-/-}$ generated by CRISPR Cas9. (c) A 986 base-pair deletion caused by CRISPR/Cas9 in the *miR-142a* locus showed failure to form mature *miR-142a-5p* and *miR-142a-3p*. (d) Predicted RNA secondary structure of wide type and mutated pri-*miR-142a* by CRISPR/Cas9. (e) *MiR-142a-3p* was absent in *142*$^{C-/-}$ embryos at 4 dpf by WISH. Red dashed circles mark the expression of *miR-142a-3p* in the thymus and red arrows mark the expression of *miR-142a-3p* in the CHT.

Supplementary Figure S2. Normal primitive hematopoiesis and vascular development in *miR-142a* knock out mutants.

(a) *142T*$^{-/-}$ embryos displayed normal expression of primitive hematopoiesis marker *scl* and erythrocyte marker *gata1* by WISH at 24 hpf. Somitic marker *myod* served as control. (b) *142*$^{T-/-}$ embryos showed normal expression of primitive myeloid marker *lyz* and *pu.1* by WISH at 24 and 36 hpf respectively. (c) Arterial marker *dll4 dltC* and *efnb2a* were unaffected in *142*$^{T-/-}$ embryos by WISH at 24 and 36 hpf respectively. (d) Venous marker *flt4* and *msr* were unaffected in *142*$^{T-/-}$ embryos by WISH at 24 and 36 hpf respectively. (e) *142*$^{T -/-}$ embryos showed normal expression of *dll4* and *flt4* at 24 and 36 hpf by qPCR (mean±SD, n=3, ns stands for no significance).

Supplementary Figure S3. Defect of HSPC emergence is specifically caused by *miR-142a-3p but not miR-142a-5p.*

(a) *142*$^{C-/-}$ embryos showed decreased expression of HSPC marker *runx1* at 26 and 36 hpf and *cmyb* at 36 hpf by WISH. (b) HSPC differentiated lineages showed reduction in *142*$^{C-/-}$ embryos including erythrocytes (*gata1*), neutrophils (*lyz*) and lymphocytes (*rag1*) at 4 dpf by WISH. (c) *MiR-142a-5p* MO injected embryos displayed normal expression of HSC marker *runx1* by WISH at 36 hpf.

Supplementary Figure S4. Analysis of the neutrophil morphology of adult *142*$^{T-/-}$ fish.

Zebrafish kidney cells were gated and sorted by FACs to collect fractions of cells including erythrocytes, lymphocytes, proerythroblasts and myeloid cells. Wright-Giemsa staining of sorted cells from *142*$^{T-/-}$ fish of 12 weeks showed normal morphology of three myeloid cell types (eosinophils, macrophages and neutrophils) compared with wide-type siblings.

Supplementary Figure S5. Increased apoptosis in *142*$^{C-/-}$ embryos and upregulated expression of apoptotic genes upon knock down of *miR-142a-3p*.

(a) TUNEL assay displayed more apoptotic cells in the AGM region of *142*$^{C -/-}$ embryos. The number was quantified according to the TUNEL positive cells in the AGM region (mean±SD, n=3, *P<0.05). (b) Microarray analysis revealed that apoptotic genes represented by different colors of graph bar including *trim50*, *p53*, *phlda3*, *caspase8*, *xrcc5*, *fas*, *sixx-35d8.1*, and *ypel3* were increased in the *miR-142a-3p* morphants at 2 and 4 dpf.

Supplementary Figure S6. *MiR-142a-3p* inhibits *p53* through the putative target sites in luciferase reporter assay and GFP reporter assay in vivo.

(a) Luciferase reporter assay showed that there was a suppression of the luciferase activity in wild-type but not mutated *p53* by *miR-142-3*p duplex in a dose dependent manner in zebrafish embryos (mean±SD, n=3, *P<0.05, ns stands for no significance). (b) The scheme of the reporter constructs of partial *p53* coding region containing the *miR-142a* binding site and GFP reporter. GFP mRNA fused with the recognition site of *p53* was co-injected with *miR-142-3*p negative control and duplex into one-cell stage embryos. GFP reporter assay showed that there was a suppression of the luciferase activity in wild-type but not mutated *p53* by *miR-142-3*p duplex in zebrafish embryos.

Supplementary Figure S7. *P53* knock down rescues excessive apoptosis and reduced differentiated populations in *142*$^{T -/-}$ embryos.

(a) *P53* MO-injected *142*$^{T -/-}$ embryos showed the restoration of excessive apoptosis at 24 hpf by TUNEL assay and statistical analysis (mean±SD, n=4,*P <0.05). (b) *P53* knock down by

p53 MO partially rescued decreased differentiated lineages in *142*$^{T-/-}$ including erythrocytes (*gata1*), neutrophils (*lyz*) and T cells (*rag1*).

Supplementary Figure S8. A model of *miR-142-3p* in controlling HSPC development. The regulation of *miR-142a-3p* in HSPC development is summarized here. In addition to the *miR-142a-3p-irf7* regulation axis, *miR-142a-3p* also acts as a repressor of *p53* by targeting its open reading frame. Loss of *miR-142a-3p* activates *p53* and leads to *p53*-dependent apoptosis and cell cycle arrest, and thus disrupts the maintenance of HSPCs.

Supplemental Tables

Table S1 The PCR primers used in qPCR assay and the sequences of *miR-142-3p* duplex and duplex control.

**Name**	**Sequence**
miR-142a-3p F	5'- GCCGCTGTAGTGTTTCCTACTT-3'
miR-142a-3p R	5'-GTGCAGGGTCCGAGGT-3'
miR-142a-5p F	5'- GCCGCCATAAAGTAGAAAGC-3'
miR-142a-5p R	5'- GTGCAGGGTCCGAGGT-3'
β-actin F	5'- GCTGTTTTCCCCTCCATTGTT-3'
β-actin R	5'- TCCCATGCCAACCATCACT-3'
u6 F	5'-TTGGTCTGATCTGGCACATATAC-3'
u6 R	5'-AAAAATATGGAGCGCTTCACG-3'
p53 F	5'- ACCACAGCTTGGTGCTGAAT -3'
p53 R	5'- GCCTGGACATGCACACACA -3'
runx1 F	5'-CGTCTTCACAAACCCTCCTCAA-3'
runx1 R	5'-GCTTTACTGCTTCATCCGGCT-3'
p21 F	5'- GACTGAGGAATGGATCTTTC -3'
p21 R	5'- CTTCATCTGTCTGGAGCTGCAT -3'
irf7 F	5'-CAAGGAACAACTGCTTAGT -3'
irf7 R	5'-GATGGTGTAATCTGGAGAC -3'
miR-142-3p duplex	5'-UGUAGUGUUUCCUACUUUAUGGACAUA AAGUAGGAAACACUACAUU-3'
miR-142-3p duplex control	5'-UUCUCCGAACGUGUCACGUUUACGUGA CACGUUCGGAGAAUU-3'

Table S2 Primers used in identifying mutants.

**Name**	**Sequence**
miR-142a TALEN WT F	5'-GTAGTGTTTCCTACTTTATGGATG-3'

miR-142a TALEN WT R	5’- TGAAAGCTTTATTAAAGTGTG-3’
miR-142a Cas9 F	5’-GTTATTGCCTCTTCCTGTG-3’
miR-142a Cas9 R	5’- ATGCGACTTGAACAGTGTAAT-3’
p53 M^{214k} WT F	5’-AGCTGATGGGGGGGAT-3’
p53 M^{214k} MU F	5’-AGCTGCATGGGGGGGAA-3’
p53 M^{214k} WT/MU R	5’-GATAGCCTAGTGCGAGCACACTCTT-3’
